# Spin injection in graphene using ferromagnetic van der Waals contacts of indium and cobalt

**DOI:** 10.1038/s41928-024-01330-w

**Published:** 2025-01-20

**Authors:** Soumya Sarkar, Saeyoung Oh, Peter J. Newton, Yang Li, Yiru Zhu, Maheera Abdul Ghani, Han Yan, Hu Young Jeong, Yan Wang, Manish Chhowalla

**Affiliations:** 1 Department of Materials Science & Metallurgy, University of Cambridge, Cambridge, UK; 2 Graduate School of Semiconductor Materials and Devices Engineering, Ulsan National Institute of Science and Technology (UNIST), Ulsan, Republic of Korea; 3 Department of Physics, Cavendish Laboratory, University of Cambridge, Cambridge, UK

**Keywords:** Materials for devices, Two-dimensional materials

## Abstract

Graphene-based spintronic devices require efficient spin injection, and dielectric tunnel barriers are typically used to facilitate spin injection. However, the direct growth of ultrathin dielectrics on two-dimensional surfaces is challenging and unreliable. Here we report spin injection in graphene lateral spin valves using ferromagnetic van der Waals contacts of indium and cobalt (In–Co), and without the deposition of dielectric tunnel barriers. With this approach, we obtain magnetoresistance values of 1.5% ± 0.5% (spin signal around 50 Ω), which is comparable to state-of-the-art graphene lateral spin valves with oxide tunnel barriers, with a working device yield of more than 70%. By contrast, lateral spin valves with non-van der Waals contacts containing only cobalt are inefficient and exhibit, at best, a magnetoresistance of around 0.2% (spin signal around 3 Ω). The contact resistance of our ferromagnetic indium–cobalt van der Waals contacts is 2–5 kΩ, which makes them compatible with complementary metal–oxide–semiconductor devices.

## Abstract (web summary)

Ferromagnetic van der Waals contacts of indium and cobalt can provide robust and reproducible spin injection in graphene lateral spin valves without the need for an additional dielectric tunnel barrier.

## Main

Creating clean metal contacts to semiconductors is central to modern electronics^1^. These metal–semiconductor heterojunctions are governed by energy-level alignments across the contact interface. However, dangling bonds and metallization induced defects lead to chemical reactions, interdiffusion and localized strain, which prevent the interface from being atomically sharp^2,3^. These issues are exacerbated for metal contacts on two-dimensional (2D) materials because atomically thin semiconductors are easily damaged during metal deposition^2,3^. Van der Waals (vdW) contacts can, however, be used to create ultra-clean interfaces with 2D semiconductors^4,5^. Such vdW contacts are characterized by the presence of a 2–4 Å vacuum gap between the metal and 2D semiconductor, and vdW contacts fabricated by mechanical transfer of metals^2^, by using metallic 2D materials^6^, and by direct metal deposition of indium alloys^4,5^ have been shown to decrease contact resistance and improve device performance.

Spin-based devices are of potential use in the development of energy-efficient computing^7–9^ and graphene has been explored for such spintronic devices because of its long spin diffusion lengths^9–11^. For efficient injection of spin-polarized electrons in lateral spin valves (LSVs), the conductivity mismatch between the ferromagnetic (FM) metal contact and graphene channel must be overcome to avoid spin backflow and interfacial spin flipping^12,13^. This is typically done by inserting thin tunnel barriers of magnesium oxide (MgO), titanium dioxide (TiO_2_) or aluminium oxide (Al_2_O_3_) between the metal contact and the spin channel^12,14^.

Graphene is, however, free of dangling bonds and therefore uniform deposition of ultrathin oxide thin films is challenging^15,16^. As a result, the yield of working graphene-based LSVs utilizing oxide tunnel barriers is low (typically less than 10%)^9,17,18^. In addition, remote interface phonon coupling and charge impurity/defect scattering at the oxide interface adversely affects the transport of spin-polarized carriers in graphene. Inserting mechanically transferred hexagonal boron nitride (h-BN) between cobalt and graphene has been shown to inject spins^19–22^. LSVs have also been created with one 2D FM Fe_5_GeTe_2_ electrode and one Co/TiO_2_ electrode on graphene^23^, which suggests that the vdW vacuum gap between 2D Fe_5_GeTe_2_ and graphene can act as a tunnel barrier for efficient spin injection.

We have previously shown that indium alloy vdW contacts on 2D materials can have a vacuum gap between the metal and the semiconductor^5^. Such indium alloy vdW contacts exhibit low contact resistance and are free from Fermi level pinning. They can also be used to create high-performance p- and n-type field effect transistors (FETs)^3–5^. In this Article, we show that vdW contacts of FM indium/cobalt (In/Co) can be fabricated by industry-compatible electron beam evaporation on graphene and can be used to inject spins. With our FM In/Co vdW contacts, we can repeatably obtain magnetoresistance (MR) values of 1.5% ± 0.5%, which is comparable to state-of-the-art graphene lateral spin valves with oxide tunnel barriers^24–26^.

## Ferromagnetic vdW contacts

**Fig. 1:**
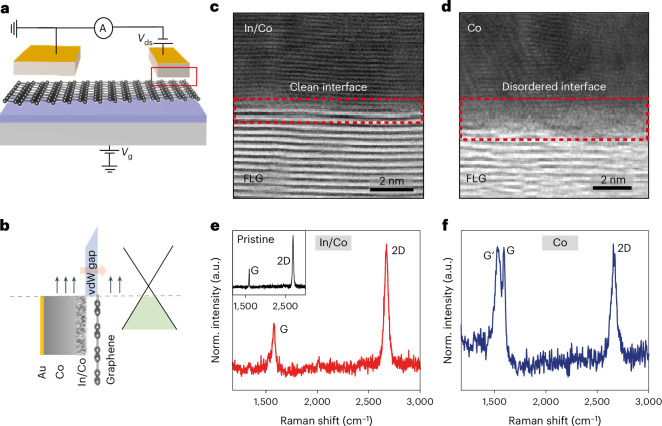
Graphene LSVs with FM vdW contacts. **a**, Schematic of a monolayer graphene LSV on SiO_2_/Si substrate with two FM vdW contacts of different widths (drain-source voltage *V*_ds_ and gate voltage *V*_g_). **b**, Schematic showing cross-section of the FM–graphene interface with their energy levels separated by a potential barrier formed by the vdW gap. The vdW contacts are ~10 nm In and ~40 nm Co, capped with ~5 nm Au. **c**,**d**, Cross-sectional ABF-STEM images of graphene/(In/Co) interface (**c**), from the red rectangle region in **a**, and the graphene/Co interface (**d**). The In/Co interface is very clean with no observable disorder. The Co/graphene interface shows damage and disorder. Few-layer graphene (FLG) was used for obtaining high-resolution images and the growth quality of In/Co metal films on FLG is similar to monolayer graphene (as shown in Supplementary Section 3). **e**,**f**, Raman spectra of In/Co on graphene (**e**) and Co on graphene (**f**). Raman spectrum is similar to pristine graphene (inset) with In/Co, while the pure Co on graphene Raman spectrum shows substantial splitting of the G band. The total thickness of the metal contact on graphene for Raman spectroscopy was limited to 20 nm to ensure optical transparency. Norm., normalized.

We fabricated LSVs (Fig. 1a,b) using mechanically exfoliated graphene and FM In/Co electrodes of dissimilar widths (and hence dissimilar coercivities) as vdW contacts for spin injection and collection (see methods for details of device fabrication). We characterized the cross-sectional interface of the In/Co contacts using high-resolution annular bright-field (ABF) scanning transmission electron microscopy (STEM). The image in Fig. 1c, which corresponds to the red rectangle in Fig. 1a, shows that In/Co contacts form a clean vdW interface with graphene. By contrast, the interface between graphene and Co (Fig. 1d) is disordered. Multiple locations of the cross-sectional interface were characterized using STEM to confirm the uniformity of In/Co metal films on graphene (shown in Supplementary Fig. 1). The energy-dispersive X-ray spectroscopy (EDS) mapping of the interface shown in Supplementary Fig. 2 indicates that the In and Co are homogeneously mixed.

Raman spectroscopy is widely used to characterize the structural quality and doping levels of graphene^27^. The Raman spectrum (Fig. 1e) of graphene measured through In/Co exhibits a single Lorentzian 2D peak at 2,671 cm^−1^ and the intensity ratio of the 2D peak to the G peak, *I*_2D_/*I*_G_ is 2.5, which suggests a doping level of ~150 meV and carrier concentration of ~10^12^ cm^−2^ (curve fitting is described in Supplementary Fig. 3). This is similar to the Raman spectrum of pristine graphene on SiO_2_ (*I*_2D_/*I*_G_ is 2.8) shown in the inset. The damage and disorder at the Co/graphene interface is confirmed by Raman via splitting of the G band and the appearance of the G′ mode (Fig. 1f) (Supplementary Section 3). Furthermore, the position of the 2D mode softens to 2,660 cm^−1^ and *I*_2D_/*I*_G_ decreases to 1.0 after Co deposition on graphene. An *I*_2D_/*I*_G_ of 1.0 corresponds to about 700 meV shift in the Fermi energy, which increases the carrier concentration to ~10^13^ cm^−2^. The large carrier density decreases the contact resistance and therefore reduces the barrier for spin injection^28^. In contrast, In/Co forms a vdW contact with graphene with a vacuum gap of ~0.3 nm (see Supplementary Section 4 for a schematic of the interface).

## Electrical transport across ferromagnetic vdW contacts

**Fig. 2:**
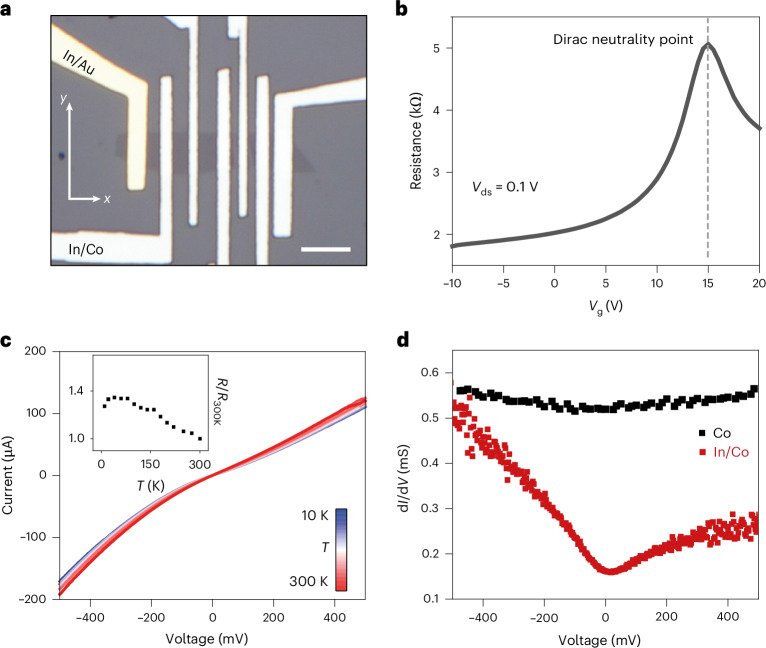
Temperature-dependent electrical transport. **a**, Optical micrograph of monolayer graphene LSV on SiO_2_ substrate with In/Co and non-magnetic In/Au contacts. Scale bar, 10 μm. **b**, Room-temperature transfer characteristics of the backgated graphene FET showing p-type carrier transport at *V*_g_ = 0 V. **c**, *I*–*V* characteristics measured from 10 K to 300 K show non-ohmic behaviour. The increase in resistance (*R*/*R*_300K_) with decreasing temperature is less than 1.4 and is almost constant below 100 K, as shown in the inset. **d**, Differential conductance versus voltage measured at 10 K supports the non-ohmic *I*–*V* characteristics for In/Co contacts (red), while pure cobalt contacts (black) suggest ohmic transport. These features suggest carrier tunnelling between FM vdW contacts and graphene.

The optical micrograph of a typical LSV device is shown in Fig. 2a, where six In/Co contacts of dissimilar widths were deposited on graphene. In/Au contacts were also included for control experiments. Graphene flakes used in this study are hole doped with a room-temperature field effect mobility of 2,000 ± 500 cm^2^ Vs^−1^ as indicated by the backgated FET transfer characteristics shown in Fig. 2b (refs. ^29,30^). The resistance at zero gate bias was found to be 2 kΩ, which is lower than graphene LSV devices with tunnel barrier contacts, in which resistances typically range from tens of kΩ to a few hundreds of MΩ (refs. ^25,31^).

Next, to understand the nature of carrier transport with In/Co vdW contacts, we performed temperature-dependent measurements. Current–voltage (*I*–*V*) measurements from 10 K to 300 K are shown in Fig. 2c. Weak temperature dependence and a non-ohmic behaviour at low bias are observed. The temperature dependence is shown in the inset, where the resistance enhancement (*R*/*R*_300K_) measured at a bias of 10 mV is <1.4 and is almost constant below 100 K. The weak temperature dependence is further corroborated by the plot of current versus 1,000/*T* in Supplementary Fig. 5a. The non-ohmic transport is also evident in the voltage dependence of differential conductance (d*I*/d*V*) at 10 K shown in Fig. 2d, where the decrease in conductance at low bias is a signature of tunnelling transport^15,32^. By contrast, the voltage dependence of d*I*/d*V* for pure cobalt contacts is relatively flat, suggesting absence of a potential barrier at the interface. The voltage dependence of conductance was fit with the Brinkman, Dynes and Rowell model^33^ for an asymmetric barrier, as shown in Supplementary Section 6, from which an effective barrier height, *φ*, of ~50 meV was extracted. The device conductance also shows very weak temperature dependence (Supplementary Fig. 5b,c), suggesting tunnelling at the FM vdW contact interface^22^. These electrical transport properties suggest field-assisted tunnelling as the dominant electron transport mechanism with FM In/Co vdW contacts^34^.

## Spin transport in graphene LSVs

**Fig. 3:**
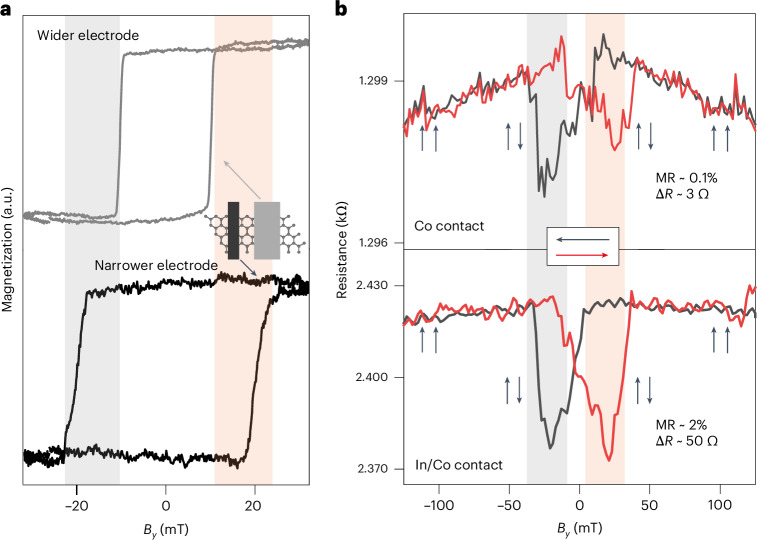
Spin transport using vdW FM contacts. **a**, Room-temperature MOKE magnetic hysteresis loops of In/Co electrodes. The top curve is for a 25 × 2 μm^2^ electrode. The bottom curve is for a 25 × 1 μm^2^ electrode. **b**, Resistance measured at 4 K while scanning in-plane magnetic field for devices with cobalt (upper panel) and In/Co contacts (lower panel), respectively. The black (red) curves represent decreasing (increasing) sweep directions of the magnetic field. The resistance changes under antiparallel spin orientation (in the shaded regions in **a** and **b**) correlate with the coercive fields of the electrodes measured by MOKE. *B*_*y*_ is the magnetic field applied parallel to the in-plane easy axis of the FM contacts.

Magneto-optical Kerr effect (MOKE) magnetometry was used to investigate the magnetic switching of In/Co electrodes at room temperature. The magnetic field was applied parallel to the in-plane easy axis of the FM contacts (along the *y* axis in Fig. 2a). The hysteresis loops (Fig. 3a and Supplementary Fig. 7) suggest uniaxial anisotropy with both contacts switching magnetization at magnetic fields of 10–20 mT. The wider electrodes show lower coercive fields (grey curve) compared to the narrow electrodes (black curve) due to shape anisotropy. From this, the magnetic field required to pin the FM contacts in parallel and antiparallel spin orientations can be estimated.

Spin transport across a non-magnetic semiconductor can be probed by measuring the change in resistance induced by change in orientation of the magnetic field (parallel to antiparallel configuration)^11^. MR characteristics of graphene LSVs were obtained by measuring the resistance of the graphene spin channel while sweeping the magnetic field in-plane (along the *y* direction parallel to the magnetic easy axis, as shown in Fig. 2a) at 4 K. The magnetic field dependence of resistance for devices with Co only and In/Co contacts is shown in Fig. 3b. As the magnetic field is decreased and increased (red and black curves), we observe two clear transitions in the device resistance. The resistance versus magnetic field characteristics shown in the upper panel of Fig. 3b from one of the few working devices with cobalt-only contacts show an MR of 0.1% (spin signal of 3 Ω). In comparison, an MR of 2% (spin signal of 50 Ω) is found in In/Co-contacted devices, as shown in the lower panel of Fig. 3b. The channel lengths for our LSVs are 2 μm, and the MR obtained is comparable to a device with a 2-μm-long spin channel made of epitaxial graphene with Co/Al_2_O_3_ tunnel barrier contacts^25^. Our In/Co device results are remarkable because no tunnel barrier is used, and they demonstrate that the vdW vacuum gap is sufficient for efficient spin injection. The measured MR is attributed to the spin valve effect, where the resistance of the spin-polarized electrons in the channel changes according to the relative spin orientation of the FM contacts and exhibits a transition during magnetization switching. The fields at which these transitions occur correspond to the coercive fields for 180° magnetization switching of the FM vdW contacts measured by MOKE (Fig. 3a), suggesting that the MR observed is due to spin transport.

The gate dependence of magneto transport (Supplementary Section 8) shows an increase in spin signal near the Dirac peak region, which provides additional evidence of tunnelling transport across the In/Co and graphene interface^26^. We have also performed four terminal, non-local spin diffusion measurements, which show MR peaks that switch at magnetic fields identical to local LSVs (Supplementary Fig. 10). We have measured the non-local spin signal from In/Co-based graphene LSVs at 10 K as a function of injection current from 1 μA to 25 μA (Supplementary Fig. 11). The linear bias dependence of the non-local signal indicates that our spin signals are not related to thermal effects^35^.

Further, we have performed MR measurements for LSVs with FM In/Co contact at one end and a non-magnetic In/Au contact at another end. These devices do not show any spin signals (Supplementary Fig. 12). All of the above results indicate that the MR observed in our graphene LSVs with In/Co contacts originates from spin-polarized transport in graphene^32^. Notably, the MR in our devices shows a decrease in resistance for antiparallel spin orientations of the contacts (Fig. 3b). Although this contradicts the standard Julliere model^36^, negative MR peaks in graphene LSV devices are not uncommon^32,36^. Negative MR can be attributed to quantum interference in the spin channel, where the sign of the MR could be related to the sign of the spin polarization at the interface between contacts and graphene^37–39^.

We have measured four terminal non-local Hanle spin precession signals for the In/Co-based graphene LSVs at 10 K, as shown in Supplementary Fig. 13, which were fitted with the Hanle spin transport equation^10^ (Supplementary Section 12) to obtain the spin lifetime and spin diffusion lengths. The spin lifetime and spin diffusion lengths for In/Co-based LSVs were found to be ~200 ps and 2.4 μm, respectively, which are similar to the values reported in the literature^15,22^. We have observed MR at room temperature, as shown in the temperature-dependent measurements of the non-local spin signal provided in Supplementary Section 13. While the spin signal and switching field are lower than at 4 K due to spin scattering, lower barrier height and temperature-dependent increase of coercivity (Supplementary Fig. 15), our results show that spin injection and transport at room temperature is possible with vdW In/Co contacts.

**Fig. 4:**
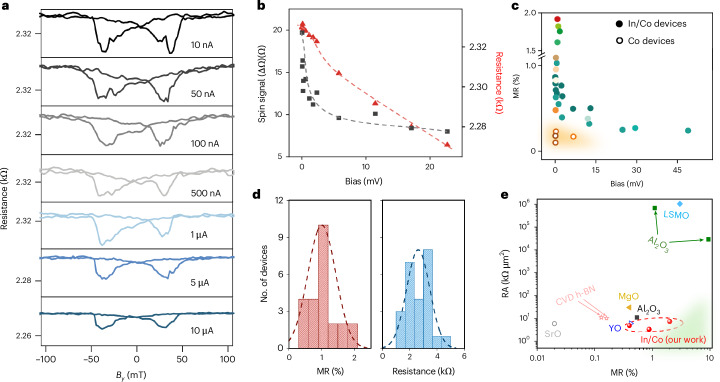
Bias-dependent magneto transport. **a**, Resistance versus in-plane magnetic field while increasing current from 10 nA to 10 μA (at 4 K). **b**, The spin signals are reduced monotonically due to bias-induced lowering of the LSV resistance. **c**, The MR values from 15 vdW In/Co devices (solid symbols) show a rapid increase as the bias is reduced. The four Co contact devices (hollow symbols) have low yield and poor MR even at low biases due to inefficient spin injection. **d**, Device statistics of MR (left) and two terminal resistances (right) measured in graphene LSVs with In/Co contacts. The dashed lines represent the normal distribution. **e**, Comparison of contact resistance and MR for LSVs with different materials for tunnel barriers from the literature^19,22,24–26,31,43,44^ with In/Co-based devices (red spheres). CVD h-BN is chemical vapor deposited hexagonal boron nitride, YO is yttrium oxide and LSMO is lanthanum strontium manganite. The solid symbols are local MR measurements while the hollow symbols are extrapolated^11^ from non-local (NL) MR measurements using Δ*R* = 2Δ*R*_NL_. *R*_NL_ is the 4-probe non-local resistance.

The magnitude of the MR in our LSVs is dependent on the applied bias, as shown in Fig. 4a. As the current sourced is increased from 10 nA to 10 μA, we see consistent spin valve signals at similar switching fields, but the MR reduces at higher current biases. Sourcing higher currents leads to an increase in the applied potential bias across the interface barrier. At higher bias, we observe a rapid decrease of the spin signal from 20 Ω to 8 Ω, while the resistance of the parallel spin state reduces only slightly from 2.34 kΩ to 2.27 kΩ (shown in Fig. 4b). Similar bias dependence has been observed in magnetic tunnel junctions and LSVs with tunnel contacts^32,40–42^.

To highlight the ease of using vdW contacts for spin injection, the MR values at different biases for 15 In/Co devices are summarized in Fig. 4c. The MR from four Co-contacted devices are also shown for comparison. For the In/Co vdW contact-based LSVs a rapid increase of MR at low bias up to 2% is observed and all our devices exhibit similar bias dependence with negligible variation. On the contrary, the MR from cobalt-based devices is lower, ≤0.2%, even at low biases (orange shaded region). Typically, we fabricate ten devices per batch. In most batches, >70% of devices show clear spin valve signals (see Supplementary Table 1 for device statistics from 23 devices). The switching fields for all our devices are similar in the range of 20–30 mT. By contrast, the yield of working devices with contacts containing only Co is substantially lower, at <10%.

The statistical distribution of MR and two terminal resistances for graphene LSVs with In/Co contacts is shown in Fig. 4d, in the form of histograms. The distribution peaks at 1.2% for MR and 2.8 kΩ for contact resistance. The low contact resistance can be attributed to the low effective barrier height resulting from the vacuum vdW gap for In/Co contacts. Such contact resistance is orders of magnitude lower than oxide or h-BN tunnel barrier contacts needed to achieve a similar MR. A comparison of MR and resistance area (RA) product of In/Co vdW contacts (shown in Fig. 4e) with previously reported spin-injection contacts based on oxide dielectrics or 2D tunnel barriers, such as h-BN, reveals that In/Co contacts possess good MR and low contact resistance.

## Conclusions

We have reported a scalable technique to deposit FM In/Co vdW contacts on graphene. The vdW contact enables robust and reproducible spin injection in graphene LSVs without the need for an additional tunnel barrier. The contact resistance in our devices is lower than in conventional LSVs. This is noteworthy because ultrahigh resistance tunnel contact devices are incompatible with complementary metal–oxide–semiconductor devices. Spintronic devices with vdW In/Co FM contacts could provide better impedance matching with low-contact-resistance FETs, faster device operation and improved spin current–energy conversion efficiency^43^, and our work highlights the potential of using a van der Waals gap as a tunnelling medium in spintronic devices.

## Methods

### Sample preparation and device fabrication

Monolayer graphene flakes were mechanically exfoliated from a high-quality bulk graphite crystal using M/H Ultron tape. The substrates were lithographically prepatterned 90/300 nm SiO_2_ on heavily doped Si, which were used as gate insulator and electrode, respectively. Monolayer graphene flakes were identified using optical microscopy, atomic force microscopy and Raman spectroscopy. The samples were coated with MMA/PMMA resist and electron beam lithography was used to pattern the electrodes. Before metal electrode deposition, the electron beam evaporation system was pumped to a base pressure of <10^−7^ Torr. Then, 8–10-nm-thick In was deposited with a low rate of 0.1 Å s^−1^ and 40-nm-thick Co was deposited subsequently. Finally, 5-nm-thick Au was deposited to prevent oxidation of cobalt. The device was rinsed with isopropanol after immersing in acetone for lift-off.

### Raman spectroscopy

Raman spectra were acquired using a Renishaw inVIA microRaman spectrometer. The samples were excited with a 514 nm laser with the power kept below 100 μW. A 2,400 lines per mm grating was used to record the spectra. For collecting the spectra from metal-coated graphene, thin layers (10–15 nm) of metals (Co, In/Co and In/Au) were deposited to ensure optical transparency.

### Transport measurements

Electrical transport characteristics were measured using a Keithley 4200 semiconductor parameter analyser system in an ambient probe station. The low-temperature measurements were performed in a Lakeshore cryogenic vacuum probe station with a closed cycle compressor and a Lakeshore temperature controller. Magneto transport measurements were performed using a Quantum Design Dynacool PPMS.

### Magneto-optic Kerr effect microscopy

Magnetic hysteresis loops were obtained from measurements of the longitudinal magneto-optic Kerr effect using a nanoMOKE 2 magnetometer, produced by Durham Magneto Optics. The laser spot was focused to a full-width at half-maximum diameter of ~5 μm and individual electrodes were located optically. The magnetic field was applied in-plane and along the long axes of the FM electrodes, parallel to their magnetic easy axis. MOKE data shown in this work are the result of taking the average of 50 individual hysteresis loops in quick succession.

### STEM sample preparation and acquisition parameters

The cross-sectional TEM specimens were fabricated using a focused ion beam (Hitachi Triple Beam NX2000). The condition of the Ga ion beam used in the last fine milling process was 3 kV, 40 pA. The damage to the sample was minimized under the conditions of low voltage and current. STEM images and EDS mapping images were taken by a FEI Titan^3^ G2 60–300 at an accelerating voltage of 200 kV. ABF-STEM images were acquired with an acceptance semi-angle of the detector range from 10 to 61 mrad.

## Supplementary information

Supplementary Information Supplementary Figs. 1–15 and Table 1.

## Data Availability

The data that support the plots within this paper and other findings of the study are available from the corresponding authors upon reasonable request.

## Code availability

The codes used for plotting the data are available from the corresponding authors upon reasonable request.
