# Supplementary material for: Spin injection in graphene using ferromagnetic van der Waals contacts of indium and cobalt
Source: Nat Electron. 2025 Jan 20;8(3):215–21. doi: 10.1038/s41928-024-01330-w (PMC11949831; doi:10.1038/s41928-024-01330-w)
Supplement: Supplementary file 1 — Supplementary Figs. 1–15 and Table 1. [file 41928_2024_1330_MOESM1_ESM.pdf]

nature electronics

Supplementary information

https://doi.org/10.1038/s41928-024-01330-w

Spin injection in graphene using ferromagnetic van der Waals contacts of indium and cobalt

In the format provided by the authors and unedited

Contents:

S1. Cross-section STEM of contact interface (Fig. S1)

S2. Energy-dispersive X-ray spectroscopy mapping of the contact (Fig. S2)

S3. Effect of metal deposition on Raman spectra of graphene (Fig. S3)

S4. Origin of vdW contacts (Fig. S4)

S5. Temperature dependent electrical transport (Fig. S5)

S6. Estimation of effective barrier height (Fig. S6)

S7. Magnetic properties of Co vs In/Co electrodes (Fig. S7)

S8. Gate dependence of MR (Fig. S8, Fig. S9)

S9. Non-local spin transport measurements (Fig. S10)

S10. Bias dependence of non-local signal (Fig. S11)

S11. Transport with non-magnetic electrode (Fig. S12)

S12. Estimation of spin lifetime (Fig. S13)

S13. Effect of temperature (Fig. S14, Fig. S15)

S14. Device statistics (Table 1)

S1. Cross-section STEM of contact interface

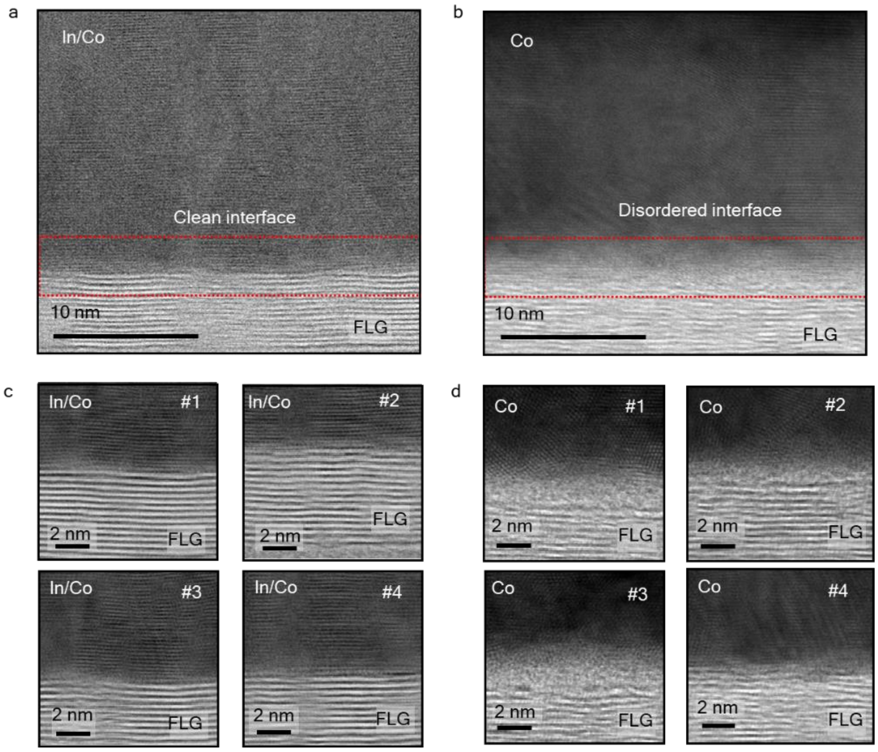

Fig. S1. Cross-sectional ABF-STEM images of (a) graphene/(In/Co) and (b) graphene/Co interface. Few-layer graphene (FLG) was used for obtaining high resolution images. Higher resolution ABF-STEM images collected from four different locations from the (c) graphene/In/Co and (d) graphene/Co interface.

S2. Energy-dispersive X-ray spectroscopy mapping of the contact

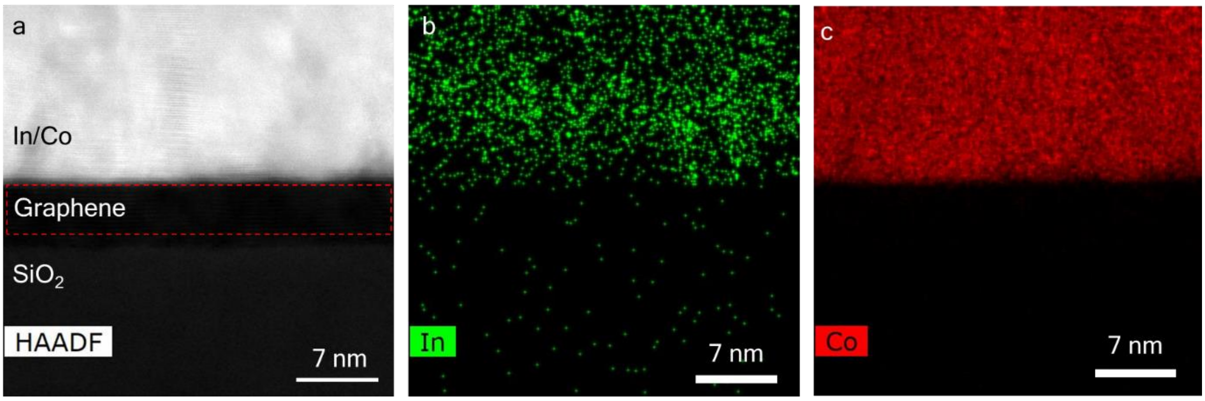

Fig. S2. (a) Cross sectional high-angle annular dark-field scanning transmission electron microscopy (HAADF-STEM) image of few layer graphene (FLG) with In/Co contact. Elemental mapping showing the distribution of (b) In and (c) Co. Co and In signals are seen to overlap in the contact region, suggesting alloying.

S3. Effect of metal deposition on Raman spectra of Graphene

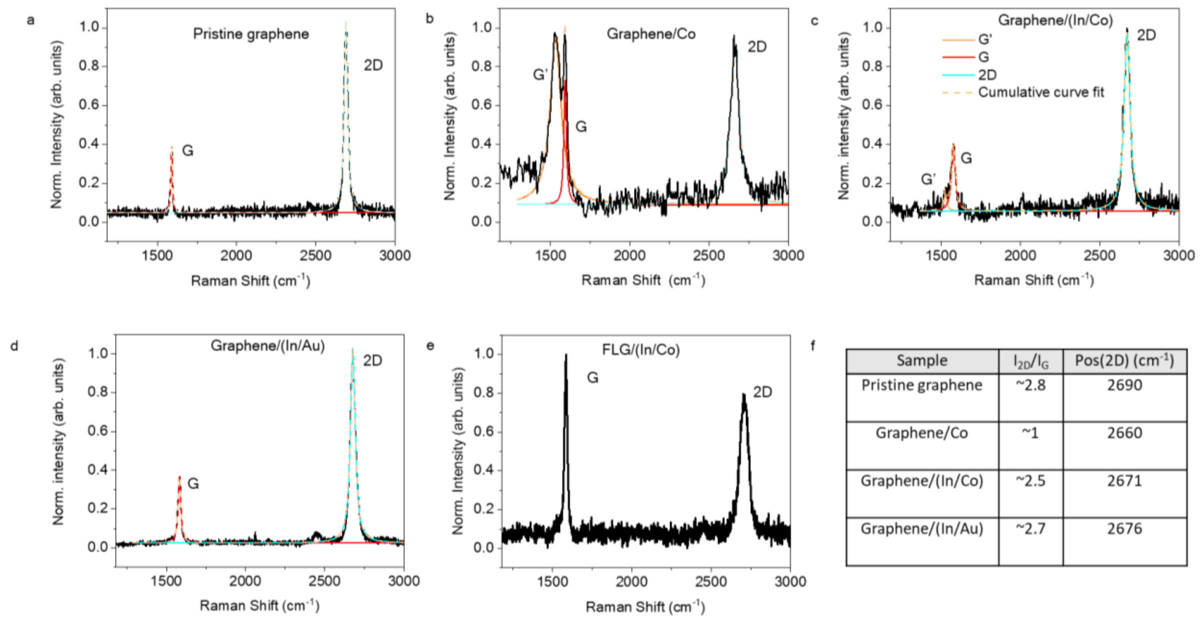

Sample	I_{2D}/I_G	Pos(2D) (cm^{-1})
Pristine graphene	~2.8	2690
Graphene/Co	~1	2660
Graphene/(In/Co)	~2.5	2671
Graphene/(In/Au)	~2.7	2676

Fig.S3. Raman spectra with the individual sub-peaks deconvoluted and fitted with a Lorentz curve for pristine graphene (a), graphene/Co (b), graphene/(In/Co), and (d) graphene/(In/Au). Deposition of Co leads to a clear splitting of the G peak to an additional G' peak, which is associated to disorder. [1] The G' peak intensity is negligible for In/Co deposition. In/Au deposition does not modify the Raman spectra when compared to pristine graphene. These results suggest that cobalt can lead to disordered interfaces with graphene. (e) Raman spectra of FLG/In/Co is similar to graphene/(In/Co) which suggests that the growth of In/Co is similar on graphene and FLG. (f) Extracted intensity ratio of 2D and G peak (I_{2D}/I_G) and position of 2D peak from Raman spectral fitting.

The G peak in the Raman spectra of graphene is attributed to the stretching of the sp^2 bonds. It corresponds to phonons near the Γ point in the Brillouin zone where the net momentum transfer is close to zero. The G' peak appears when graphene is doped and has been attributed to (i) phonon-induced intra-band electronic transitions, and (ii) softening of the G mode phonons due to hybridization between p-orbitals in graphene and d-orbitals in cobalt.[1] To conclude, the G' peak confirms chemisorption and charge-transfer between cobalt and graphene.

S4. Origin of vdW contacts

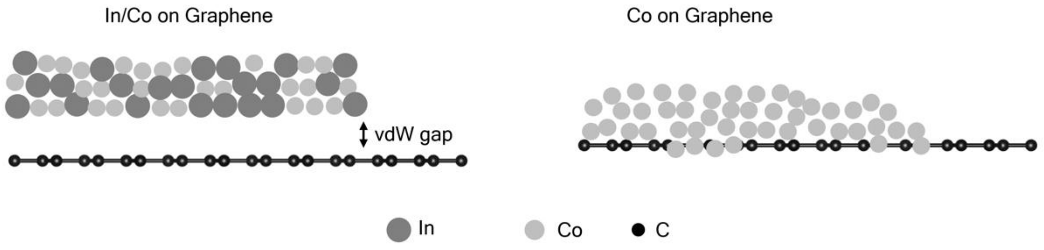

Fig. S4. Schematic of the atomic structure of the interface between In/Co and graphene that forms a vdW contact, and Co and graphene that forms a rough and defective interface.

Indium alloy based vdW contacts on 2D materials can be deposited at lower evaporation power.[2] As a result, the interface between In/Co and graphene is sharp without defects, and can be described by van der Waals interactions across a vacuum gap (thickness ~0.3 nm), (Fig. S3). The vdW vacuum gap can act as a tunnel barrier for efficient spin injection. However, cobalt due to its high chemical reactivity chemisorbs and forms a rough and defective interface.

S5. Temperature dependent electrical transport

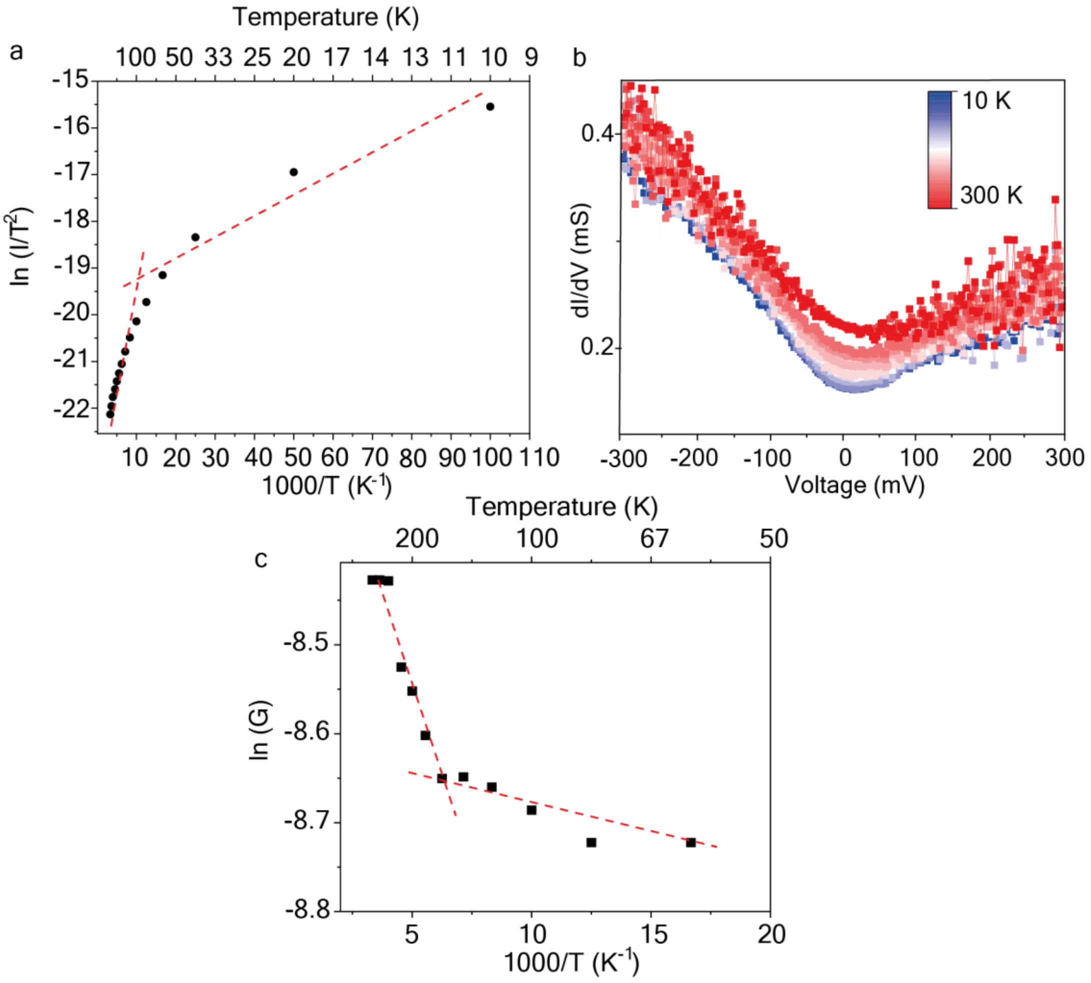

Fig. S5. The transport properties described here are for the sample in Figure 2. (a) $\ln(I/T^2)$ vs 1000/T plot shows a positive slope, which indicates tunnelling transport across the entire temperature range. The current (I) is collected at 100 mV. The slope shown by the red dashed line shows a change around 100 K, above which it increases, suggesting thermally assisted tunnelling.[3] (b) dI/dV versus voltage from 10 K to 300 K shows weak temperature dependence. (c) Temperature dependence of logarithmic conductance at 10 mV shows a gradual increase in conductance till 150 K, above which it increases rapidly due to onset of thermally assisted tunnelling.[3] Red dashed line is a guide to the eye.

S6. Estimation of effective barrier height

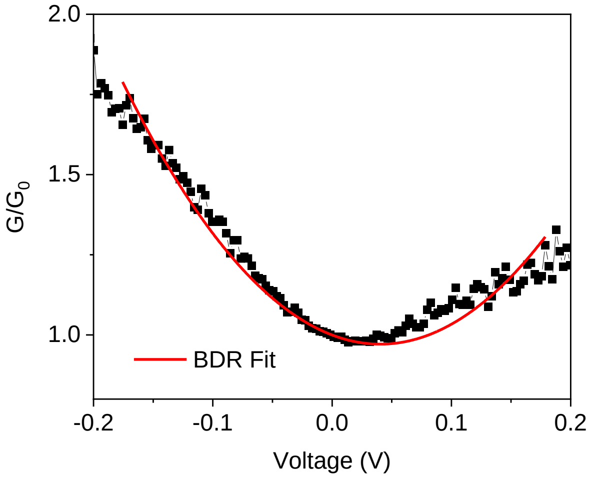

Fig. S6. Conductance at 10 K normalized by zero bias conductance (G/G_0). The red line is a fit according to the BDR model. [4]

To estimate the effective barrier height (φ) caused by the vdW gap at the interface of graphene and In/Co electrodes we use the Brinkman, Dynes and Rowell (BDR) model for tunnelling conductance of asymmetrical barriers which suggests:

$$\frac{G}{G_0} = 1 - \left(\frac{A_0 \Delta\varphi}{16\varphi^{\frac{3}{2}}}\right) eV + \left(\frac{9}{128}\frac{A_0^2}{\varphi}\right)(eV^2), \qquad (1)$$

Where φ_1 and φ_2 are barrier heights at the two sides of the trapezoidal tunnel barrier, $\Delta\varphi = \varphi_2 - \varphi_1,$ $A_0 = 4\,(2m)^{\frac{1}{2}} d/3\hbar$, m is mass of the electron, d is the barrier thickness assumed to be ~ 3 Å in our case.

Fitting the conductance in Fig. S4 (red curve), with equation (1), we can extract $\varphi = 50\ meV$ and $\Delta\varphi = 73\ meV$

S7. Magnetic properties of In/Co vs Co electrodes

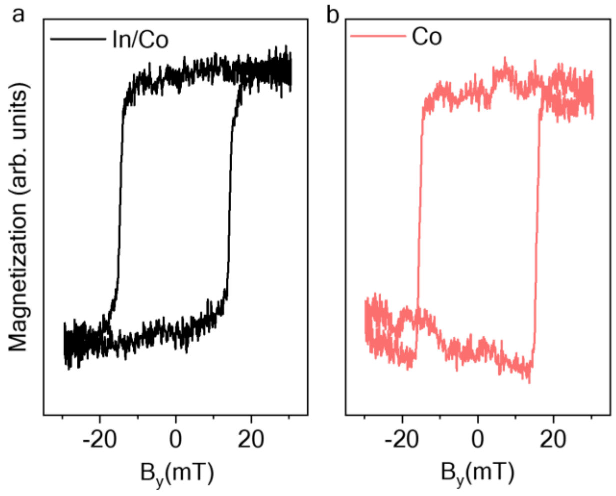

Fig. S7. Room temperature MOKE magnetic hysteresis loops of In/Co electrodes compared with Co electrodes show similar coercive fields.

S8. Gate dependence of MR

We have measured the gate dependence of resistance while scanning in-plane magnetic field as shown in Fig. S7. The gate dependence of the spin signal (ΔR) at 10 K is plotted in Fig. S8b. The spin signal increases from ~2.5 Ω in the saturation region to ~5 Ω near the Dirac peak region (Fig. S8). The observed gate dependence of ΔR provides additional evidence of tunnelling transport across the In/Co and graphene interface. As described by Han et al., spin injection across a tunnel barrier interface produces a spin dependent chemical potential $\Delta\mu = \mu_{\uparrow} - \mu_{\downarrow} = eP_J R_G I$, where R_G is the resistance of the graphene channel.[5] As we approach the Dirac point, R_G and therefore Δμ increases. This results in a peak in the observed spin signal close to the Dirac point.

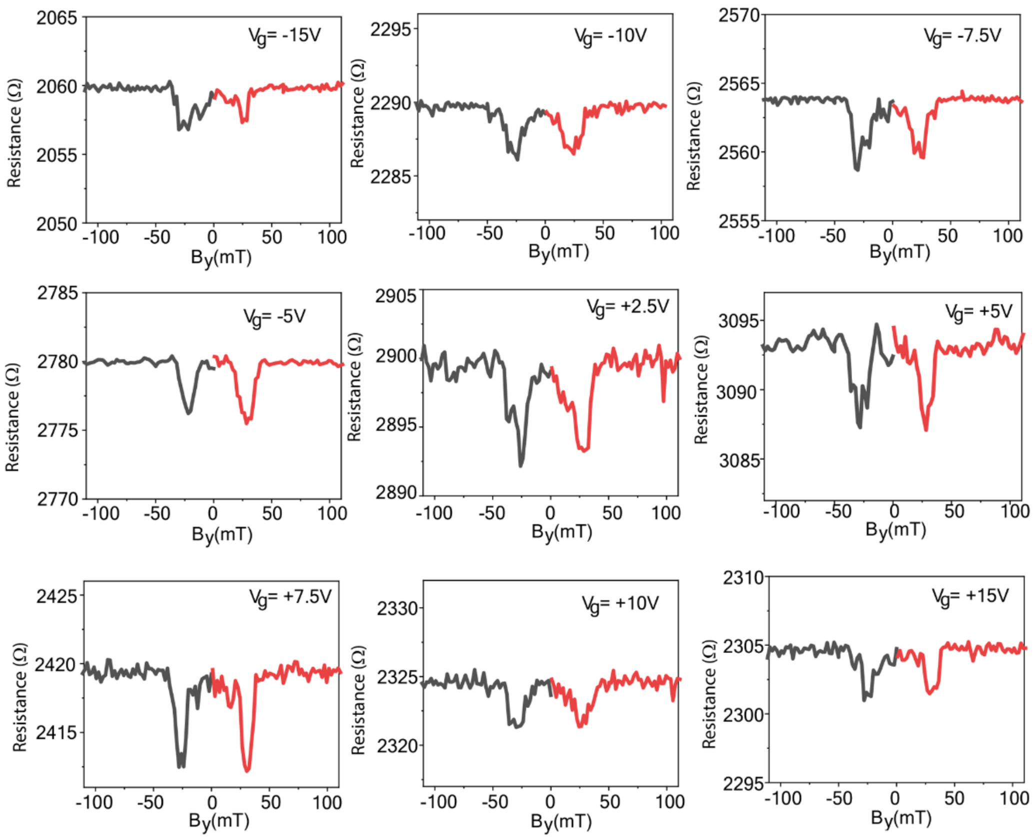

Fig. S8. Resistance of In/Co based graphene LSVs measured at 10 K while scanning in-plane magnetic field. The black (red) curves represent decreasing (increasing) sweep directions of magnetic field. The resistance changes in the antiparallel spin orientation. For clarity, we only plot the data when the magnetic field has crossed zero value. The injection current was 5 μA.

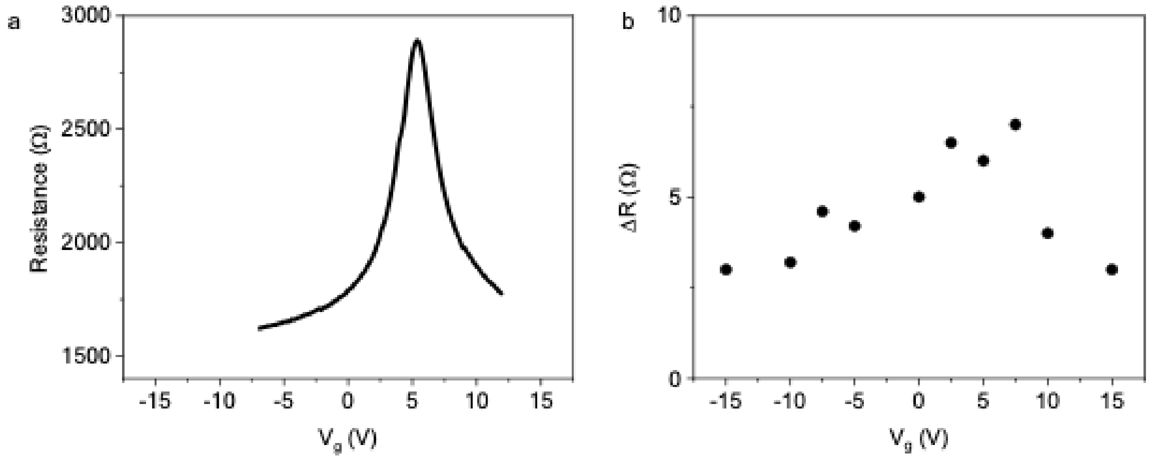

Fig. S9. (a) Room temperature resistance vs gate voltage characteristics of a graphene FET with In/Co contacts. (b) The change in spin signal (ΔR) as a function of gate voltage.

S9. Non-local spin transport measurements

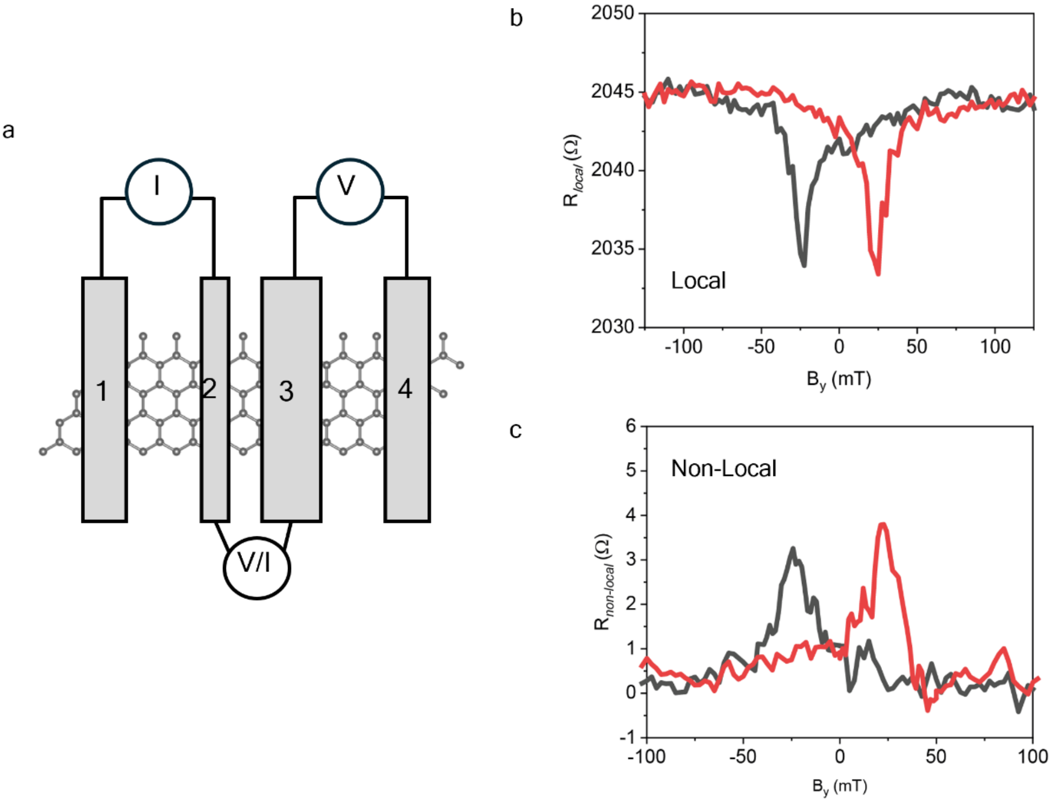

Fig. S10. (a) Schematic of a typical 4-terminal graphene LSV with In/Co contacts. The first pair of contacts (1-2) are used to source spin injection currents and the second pair (3-4) are used to detect the non-local spin signal. The same device was also used to measure local spin signal, where both spin injection and collection is measured across the same pair of electrodes (2-3) Both the (b) local and (c) non-local resistances show changes when the spin injector and collector are in antiparallel magnetization orientation. The source current for both measurements is 10 μA. The observation of non-local spin signals due to spin diffusion from electrode 2 to electrode 3 suggests that the MR peaks observed in In/Co graphene LSVs are due to spin-polarized transport of carriers through graphene. [6]

S10. Bias dependence of non-local spin signal

We have measured the non-local spin signal from In/Co based graphene LSVs at 10 K as a function of injection current from 1 μA to 25 μA (see Fig. S10). The linear bias dependence of the non-local signal indicates that our spin signals are not related to thermal effects. Spin current due to thermal spin injection from a ferromagnet is driven by the spin Seebeck

coefficient ($S_{\uparrow\downarrow}$), and change in temperature (∇T) of the contact, expressed as $J_{Thermal\,(\uparrow\downarrow)} \approx S_{\uparrow\downarrow}.\nabla T$. The temperature change is attributed to joule heating and hence, spin signals due to thermal effects should scale quadratically with injection current ($\nabla T \propto I^2$).[7]

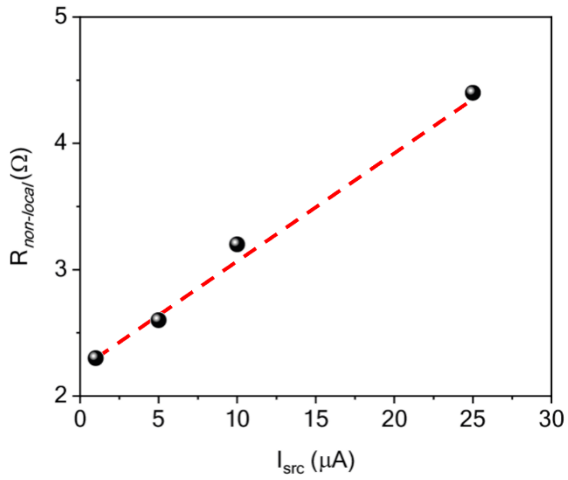

Fig. S11: Change of the non-local spin signal as a function of injection current measured at 10 K for an In/Co based graphene LSV.

S11. Transport with non-magnetic electrode

If the magnetoresistance (MR) peaks observed in our LSVs were due to spurious effects such as anisotropic magnetoresistance which occur due to non-180° switching of magnetization, it should be observed even in devices with a single ferromagnetic (FM) contact.[8, 9] To verify this we have fabricated non-magnetic (NM) In/Au electrodes on our devices as shown in the schematic in Fig. S12a. As we measure the resistance between FM In/Co and NM In/Au electrodes while sweeping magnetic field in-plane, we do not observe any spin-valve signals under the same experimental conditions as shown in Fig. S12b. This suggests that the MR peaks observed in our LSVs can be attributed to spin-polarized transport through graphene.

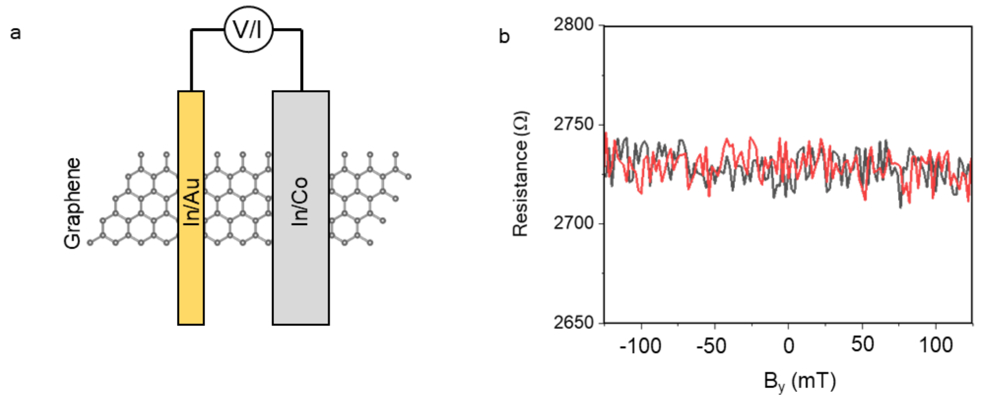

Fig. S12. (a) Schematic of the device with a FM and a NM electrode (b) Two-terminal resistances measured at 4K while scanning in-plane magnetic field for a test device with one FM In/Co and another NM In/Au electrode. The black (red) curves represent the downward (upward) sweep directions for the magnetic field. No spin valve signals are observed.

S12. Estimation of spin lifetime

We have measured four terminal non-local spin signals for the In/Co based graphene LSVs at 10 K as shown in Figure S12a. The corresponding non-local Hanle spin precession signal in Fig. S12b was fitted with the Hanle spin transport equation[10] (Eq.1) to obtain the spin-lifetime and spin diffusion lengths.

$$V_{NL} \propto \pm \int_0^{\infty} \frac{1}{\sqrt{4\pi Dt}} e^{-\frac{L^2}{4Dt}} \cos(\omega_L t) e^{-\left(\frac{t}{\tau_s}\right)} dt \qquad (1)$$

Where $\omega_L = \frac{g\mu_B B_z}{\hbar}$, is the Larmor frequency, that can be calculated as the values of $\hbar$, μ_B, and g are known (g=2 for graphene). The spin relaxation time was found to be $\tau_s = 200$ ps, and diffusion constant of D = 0.03 m^2s^{-1}. A spin relaxation length of 2.4 μm was calculated as $\lambda_s = \sqrt{D\tau_s}$. Spin lifetime measurements are sensitive to device geometry and our values are comparable to previous reports for exfoliated graphene LSVs with 1–2-layer h-BN tunnel contacts[10] and LSVs with AlOx contacts.[11]

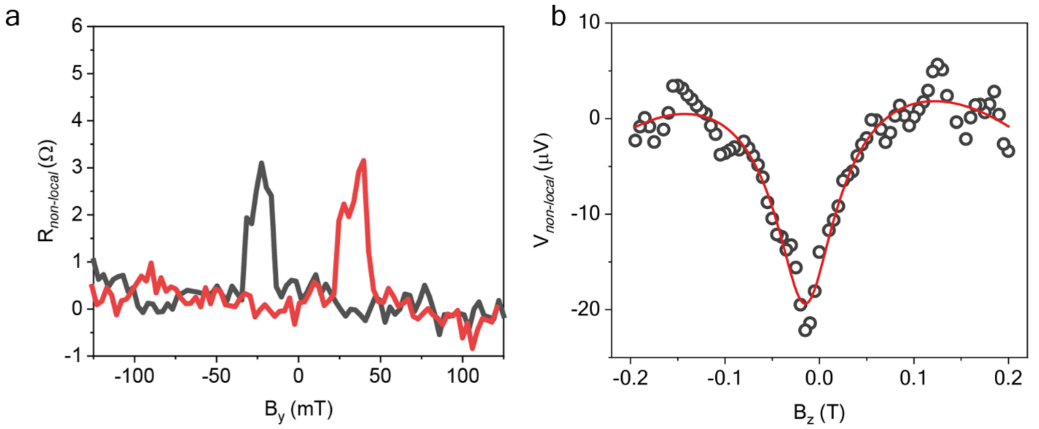

Fig. S13. (a) Four-terminal non-local spin signal measured at 10 K for an In/Co based graphene LSV while scanning in-plane magnetic field using an injection current of 5 μA (b) Non-local Hanle spin precession signal measured as a function of perpendicular magnetic field, keeping magnetization of the ferromagnetic electrodes in parallel in-plane configuration.

S13. Temperature dependent magneto transport

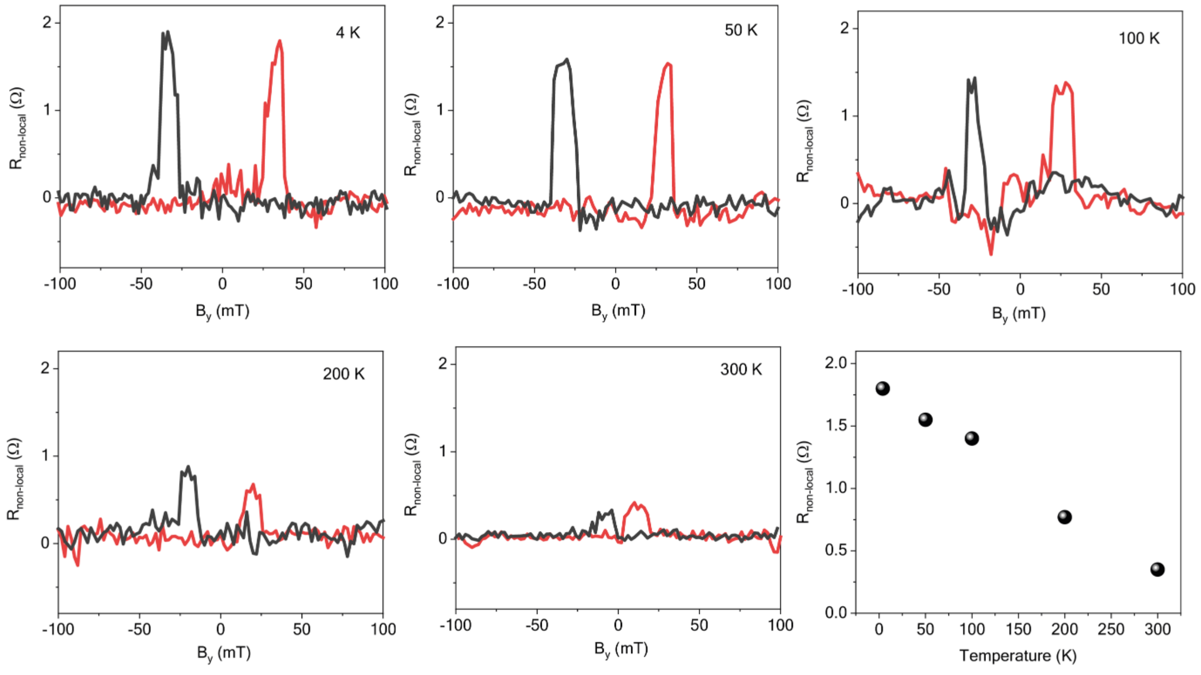

Fig. S14. Four terminal non-local resistance measured from 4 K to 300 K while scanning in-plane magnetic field for graphene LSVs with In/Co contacts. The black (red) curves represent decreasing (increasing) sweep directions of magnetic field. The non-local resistance reduces with increasing temperature, which is due to the reduction of contact resistance above 150 K, (as shown in inset of Figure 2c of main text), and onset of scattering mechanisms at higher temperature.

Temperature dependent four-terminal non-local MR measurements measured at a source current of are shown in Fig. S13. As the temperature is increased from 4 K to 300 K the non-local spin signal reduces from 1.5 Ω to 0.2 Ω. Thus, In/Co based FM vdW contacts can inject spins at room temperature. However, the injection efficiency reduces as the barrier height and resulting contact resistance reduces at temperatures above 150 K (consistent with inset of Figure 2c in main text). Additionally, at higher temperatures, spin scattering due to defects and phonons are activated, reducing the spin signal.

The coercive fields of the electrodes are expected to increase slightly at lower temperatures due to changes in magnetic anisotropy of domains as evident by the wider hysteresis observed in the MR at 4 K (Fig. 3b in the main manuscript). From the temperature dependence of the switching fields of the non-local spin signal as discussed above in Fig. S13, we have extracted the coercive fields of the wider and narrower contacts as shown in Fig. S14 below. As expected, the coercive fields for both contacts increase as temperature is lowered from 300 K to 4 K.

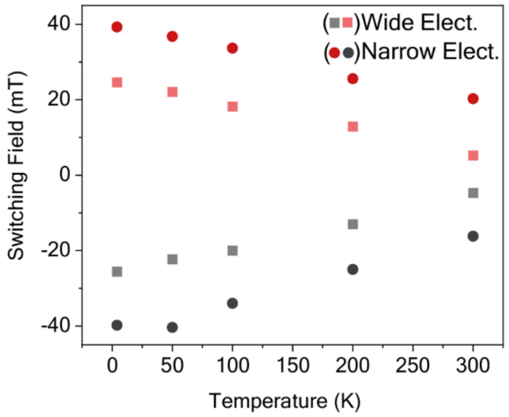

Fig. S15. Change of switching fields of the wide and narrow electrode as a function of temperature.

S14. Device statistics

This section compares the device performance for a batch of In/Co based LSVs with pure Co based LSVs. For the In/Co batch, 32 devices were fabricated out of which 23 showed clear MR (>70% of devices work). For pure Co, only 4 devices out of 20 showed MR like signals, with only 2 of them having MR peaks in both positive and negative magnetic fields (<20% of devices work).

Table 1: The resistance, spin signal and MR for 23 devices based on In/Co from a batch of 32 working devices, and 4 devices from a batch of 20 based on Co have been summarized. The applied magnetic field at which the maxima of the MR peaks are observed (defined as B_{MR}) are also tabulated.

**In/Co Contacts**				
Device	Resistance (Ω)	|Spin Signal(Ω)|	MR	B_{MR} (mT)
Current: 10 nA				
#1	1506	25	1.6%	-24/+25
#2	1906	25	1.3%	-20/+20
#3	2675	23	0.9%	-21/+20
#4	4459	41	0.9%	-32/+35
#5	1887	20	1%	-25/+24
Current: 100 nA				
#5	2495	25	1.0%	-21/+18
#6	2527	28	1.1%	-22/+25
#7	3032	24	0.8%	-26/+22
#8	3715	34	0.9%	-30/+28
#9	2420	50	2.0%	-21/+21
#10	3600	60	1.7%	-26/+30
#11	3590	42	1.2%	-30/+31
#12	3620	65	1.8%	-32/+35
#13	3052	30	1.0%	-22/+22
#14	2075	20	1%	-24/+23
#15	2328	16	0.7%	-31/+28
#16	1497	14	1%	-35/+37

Current: 500 nA				
#17	1350	14	1.0%	-25/+25
#18	3025	25	0.8%	-25/+25
Current: 1 μA				
#19	3585	25	0.8%	-35/+34
#20	1989	8	0.4%	-36/+35
#21	1826	7	0.4%	-32/+32
#22	1748	8	0.5%	-29/+28
#23	3068	12	0.4%	-25/+29
Co Contacts				
Device	Resistance (Ω)	Spin Signal (Ω)	MR	B_{MR} (mT)
Current: 100 nA				
#1	695	-1	0.1%	-13/+4
#2	1297	-1.3	0.1%	-25/+23
#3	1095	-2	0.2%	n.a./+13
#4	2605	-3	0.1%	n.a./+11

References

1 Serrano-Esparza, I. *et al.* The nature of graphene–metal bonding probed by Raman spectroscopy: the special case of cobalt. *Journal of Physics D: Applied Physics* **49**, 105301 (2016).

2 Wang, Y. *et al.* Van der Waals contacts between three-dimensional metals and two-dimensional semiconductors. *Nature* **568**, 70-74 (2019).

3 Omar, S. & van Wees, B. J. Graphene-WS 2 heterostructures for tunable spin injection and spin transport. *Physical Review B* **95**, 081404 (2017).

4 Brinkman, W., Dynes, R. & Rowell, J. Tunneling conductance of asymmetrical barriers. *Journal of applied physics* **41**, 1915-1921 (1970).

5 Han, W. *et al.* Tunneling spin injection into single layer graphene. *Physical review letters* **105**, 167202 (2010).

6 Tombros, N., Jozsa, C., Popinciuc, M., Jonkman, H. T. & Van Wees, B. J. Electronic spin transport and spin precession in single graphene layers at room temperature. *nature* **448**, 571-574 (2007).

7 Slachter, A., Bakker, F. L., Adam, J.-P. & van Wees, B. J. Thermally driven spin injection from a ferromagnet into a non-magnetic metal. *Nature Physics* **6**, 879-882 (2010).

8 Wang, W. *et al.* Magnetotransport properties of mesoscopic graphite spin valves. *Physical Review B* **77**, 020402 (2008).

9 Xu, J. *et al.* Spin inversion in graphene spin valves by gate-tunable magnetic proximity effect at one-dimensional contacts. *Nature communications* **9**, 1-6 (2018).

10 Kamalakar, M. V., Dankert, A., Bergsten, J., Ive, T. & Dash, S. P. Enhanced tunnel spin injection into graphene using chemical vapor deposited hexagonal boron nitride. *Scientific reports* **4**, 6146 (2014).

11 Han, W., Kawakami, R. K., Gmitra, M. & Fabian, J. Graphene spintronics. *Nature nanotechnology* **9**, 794-807 (2014).
